# The Relation of Skeletal Malocclusion and Airway Volumes: A Cross-Sectional Study

**DOI:** 10.1155/ijod/2318588

**Published:** 2025-09-05

**Authors:** AmirHossein SohrabiFar, Donya Maleki, Arayeh Maleki, Helia Zare, Dina Maleki

**Affiliations:** ^1^Dental Sciences Research Center, School of Dentistry, Guilan University of Medical Sciences, Rasht, Iran; ^2^Dental Sciences Research Center, School of Dentistry, Tehran University of Medical Sciences, Tehran, Iran

**Keywords:** CBCT, malocclusion, orthodontics, pharyngeal airway volume

## Abstract

**Aim:** This study evaluated the relationship between airway volume and skeletal malocclusion.

**Methods:** This study was a cross-sectional analytical study obtaining 450 cone-beam computed tomography (CBCT) images from the archives of a private clinic taken by the Sirona Galileos Comfort Plus, Dentsply Sirona, Germany device, following the Fast-Scan protocol with 14 s exposure time, FOV = 15 × 15 cm, kV = 98, and mA = 3. The CBCT images were from adults aged 17–39 years with a normal pattern in their vertical growth (SN-GO.GN = 32 ± 5), with no history of orthognathic or rhino surgery, no syndromes, no previous trauma, and no pathologies along the airway and pharynx detectable in the images. CBCT images with radiographical artifacts and low quality or resolution were excluded from the study. The total pharyngeal volume (TPV) was measured from the superior part of the PNS (posterior nasal spine) parallel to the standard horizontal plane to the anterior–inferior part of the C4 vertebra, parallel to the standard horizontal plane. Velopharynx volume (VPV) was measured from the superior part of the PNS to the inferior border of the soft palate. Glossopharynx volume (GPV) was measured from the inferior border of the soft palate to the superior tip of the epiglottis. The volumes were reported in mm^3^. To analyze data, SPSS version 22.0 (IBM Corp, Armonk, NY, USA) was used. ANOVA and Tukey's post hoc test were applied.

**Results:** The results revealed that the mean total pharyngeal airway volume and velopharyngeal airway volume were significantly larger in Class III patients compared to Class II and Class I patients. Also, in Class I patients, the mean total pharyngeal airway volume and velopharyngeal airway volume were significantly greater than in Class II patients. The glossopharynx airway volume was significantly different between Class II and Class III patients, so the glossopharynx airway volume was significantly greater in Class III patients than in Class II patients.

**Conclusion:** The results showed that there is a relationship between skeletal malocclusion and airway volumes.

## 1. Introduction

Dental occlusion plays a significant role in both orthodontics and craniofacial development. One of the most enduring methods for classifying occlusions is Angle's classification, introduced by Edward Angle in 1899. This system categorizes occlusal relationships into three distinct classes: Class I (normal occlusion), Class II (retrognathism), and Class III (prognathism), based primarily on molar positioning [1, 2]. Over the years, this classification has not only been pivotal for diagnosing malocclusions but has also become essential in studying their broader effects, including their impact on airway volume [3].

Malocclusions are quite common, with Class I occlusion representing the majority, affecting ~50%–60% of the population. Class II malocclusions, involving a retrusive mandible, affect around 25%–30%, while Class III malocclusions, characterized by a protrusive mandible, are found in 5%–10% of individuals [4–6]. Although Class I is generally considered favorable, minor misalignments within this class may still lead to functional problems. However, Class II and III malocclusions are more frequently related to more serious health concerns, particularly regarding airway obstruction [7].

Recent research has highlighted the connection between occlusion types and pharyngeal airway volume, particularly in Class II malocclusion, which has been linked to a narrower airway in the oropharyngeal and nasopharyngeal regions [8–10]. This reduction in airway volume can contribute to conditions such as obstructive sleep apnea (OSA), where the airway is blocked partially or completely during sleep, causing significant health risks [11, 12]. On the other hand, patients with Class III malocclusion may have a wider airway, although the relationship is more complex and influenced by individual anatomical differences [13].

In light of these findings, our study seeks to explore how Angle's occlusion classification affects airway space volume, using cone-beam computed tomography (CBCT), provides more precise measurements than traditional cephalometric techniques [14]. By examining airway dimensions across different occlusion classes (Classes I, II, and III), we aim to better understand the impact of malocclusion on airway size. This could have significant implications for managing airway-related issues, especially in those at risk of OSA [15–17].

Previous studies have demonstrated that Class II malocclusion is often related to smaller airway dimensions, especially in the nasopharyngeal region [10–19]. As the use of CBCT becomes more prevalent in orthodontics, understanding these relationships will be crucial in designing treatments that not only address dental alignment but also improve airway health [20–22]. This study evaluated the volume of the airway and its relationship with skeletal malocclusion to address the gap in comparative volumetric data across all three skeletal malocclusion classes (Classes I, II, and III) using standardized CBCT methods. Prior research has often focused on specific malocclusion classes or used inconsistent measurement techniques, limiting comprehensive comparisons of airway volumes. By employing a standardized CBCT protocol (Sirona Galileos Comfort Plus, Fast-Scan, 14 s exposure, FOV = 15 × 15 cm, kV = 98, mA = 3) and precise anatomical landmarks for measuring total pharyngeal volume (TPV), velopharynx volume (VPV), and glossopharynx volume (GPV), this study provides a robust analysis of airway volume differences across all three classes in a sample of 450 adults with normal vertical growth patterns. This approach enhances the understanding of how skeletal malocclusion influences airway dimensions, particularly in relation to potential health risks like OSA.

## 2. Materials and Methods

This cross-sectional analytical study used 450 CBCT images from the archives of a private clinic taken by the Sirona Galileos Comfort Plus, Dentsply Sirona, Germany device, following the Fast-Scan protocol with 14 s exposure time, FOV = 15 × 15 cm, kV = 98, and mA = 3.

The included 450 CBCT images were from adults aged 17–39 years with a normal pattern of vertical growth (SN-GO.GN = 32 ± 5), with no history of orthognathic or rhino surgery, syndromes, traumatic experience, or any pathologies along the airway and pharynx detectable in the CBCT images. CBCT images with radiographical artifacts and low quality or resolution were excluded from the study.

Radiographic artifacts were defined as distortions compromising diagnostic quality, including motion artifacts (blurring or double contours due to patient movement), metal-induced artifacts (streaks or dark bands from metallic restorations), beam hardening artifacts (dark bands from dense structures), ring artifacts (concentric rings from detector errors), scatter artifacts (noise from scattered X-rays), and aliasing artifacts (jagged edges from insufficient sampling).

Poor image quality was characterized by low resolution (pixelated or blurry images hindering anatomical landmark identification), inadequate contrast (poor differentiation between airway and soft tissues), high noise levels (grainy images from low signal-to-noise ratio), incomplete FOV (failing to capture the airway from posterior nasal spine to C4 vertebra), or improper patient positioning (deviation from the standard horizontal plane).

Prior to the study, two researchers underwent training and calibration for artifact and image quality assessment. For this purpose, 30 CBCT images were provided. Also, a detailed manual with visual examples of artifacts and image quality issues was provided for the researchers. The researchers assessed the artifact and image quality of the provided 30 CBCT images. Then, interobserver agreement for artifact and image quality evaluation was assessed using Cohen's kappa, achieving a value greater than 0.8.

Also, prior to the study, the same two researchers were trained and calibrated to determine the skeletal pattern on CBCT images and to measure the TPV, VPV, and GPV in three-dimensional (3D) images. For this purpose, 30 CBCT images and 30 3D images were provided. A detailed manual was provided with visual examples of skeletal pattern determination and airway volume measurements. The two researchers determined the skeletal pattern and measured the airway volumes. Then, interobserver agreement for skeletal pattern determination and airway volume measurements was assessed using Cohen's kappa, achieving a value greater than 0.8.

For this study, the two calibrated researchers assessed 450 CBCT images. The images with artifacts and poor image quality were excluded. Skeletal pattern was determined in the remaining images that satisfied the inclusion criteria as follows:

To determine the skeletal pattern, the ANB angle was measured using lateral cephalometric images extracted from the CBCT images. Based on these measurements, the samples were categorized as three groups: Class I malocclusion (ANB angle between 1° and 4° and Wits between 0 and −1), Class II (ANB angle greater than 4° and Wits greater than 1), and Class III (ANB angle less than 0° and Wits less than −1). The samples were enrolled if the patients had a mandibular plane angle of SN-GO.GN = 32 ± 5.

After acquiring the CBCT images, they were converted to DICOM format and imported into Dolphin 3D software. The total pharyngeal airway volume, each section's volume, and the minimum cross-sectional area (the most constricted point of the airway) were evaluated.

**Figure 1:**
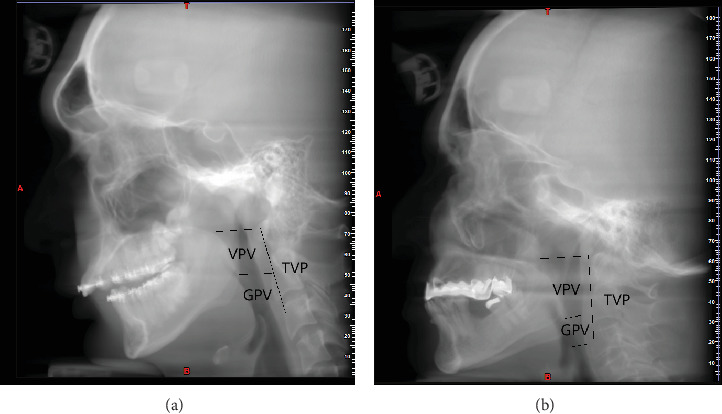
TVP, VPV, and GPV of Class II (A) and Class III (B) patients in a CBCT image.

The TPV was measured from the superior part of the PNS (posterior nasal spine) parallel to the standard horizontal plane to the anterior-inferior part of the C4 vertebra, parallel to the standard horizontal plane. VPV was measured from the superior part of the PNS to the inferior border of the soft palate. GPV was measured from the inferior border of the soft palate to the superior tip of the epiglottis. The volumes were reported in mm^3^ (Figure 1).

To analyze the data, SPSS version 22.0 (IBM Corp, Armonk, NY, USA) was used, and ANOVA and Tukey's post hoc test were applied to determine the relation of airway dimensions and skeletal classification. A *p*-value of < 0.05 was considered statistically significant.

## 3. Results

**Figure 2:**
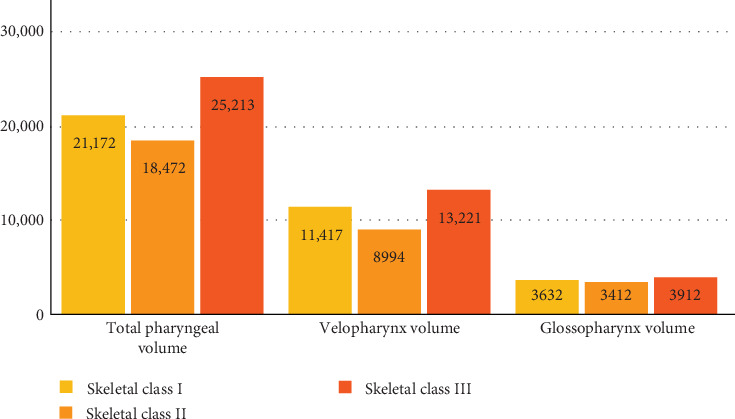
The means of TPV, VPV, and GPV in the study groups (mm^3^).

Data from all participants (*n* = 450) were analyzed, with the sample distributed across three skeletal malocclusion groups: Class I (*n* = 150), Class II (*n* = 150), and Class III (*n* = 150). The means and standard deviations of TPV, VPV, and GPV for each group are visualized in Figure 2.

ANOVA analysis revealed significant differences in TPV, VPV, and GPV among the study groups (*p*=0.001, *p*  < 0.001, and *p*=0.001, respectively). Specifically, the mean TPV was highest in Class III patients (28,500 ± 3200 mm^3^), followed by Class I (25,213 ± 2900 mm^3^), and lowest in Class II (18,472 ± 2600 mm^3^). Tukey's post hoc test confirmed that TPV in Class III was significantly greater than in Class I (*p*=0.002) and Class II (*p*  < 0.001), and Class I TPV was significantly greater than Class II (*p*=0.005). For VPV, Class III patients exhibited the highest mean volume (13,221 ± 1500 mm^3^), followed by Class I (11,417 ± 1300 mm^3^), and Class II (8994 ± 1200 mm^3^). Tukey's post hoc test showed significant differences between Class III and Class I (*p*=0.043), Class III and Class II (*p*  < 0.001), and Class I and Class II (*p*=0.034). For GPV, Class III patients had a mean volume of 3912 ± 1100 mm^3^, significantly greater than Class II (3412 ± 1000 mm^3^, *p*  < 0.001), but not significantly different from Class I (3632 ± 1000 mm^3^, *p*=0.156). No significant difference was found between Class I and Class II for GPV (*p*=0.108).

These results indicate that Class III malocclusion is associated with significantly larger airway volumes (TPV and VPV) compared to both Classes I and II, while Class I patients have larger TPV and VPV than Class II patients. For GPV, the significant difference was observed only between Classes III and II, suggesting that the glossopharyngeal region may be less influenced by skeletal malocclusion class compared to the total and velopharyngeal regions.

## 4. Discussion

This study evaluated the relationship between skeletal malocclusion and airway volumes using CBCT in 450 adults aged 17–39 years with normal vertical growth patterns. The key findings indicate significant differences in airway volumes across skeletal malocclusion classes (Classes I, II, and III). Specifically, the mean TPV and VPV were significantly greater in Class III patients compared to both Classes II and I patients. Additionally, Class I patients exhibited significantly larger TPV and VPV compared to Class II patients. The GPV was significantly larger in Class III patients than in Class II patients, with no significant difference between Class I and either Class II or III.

### 4.1. Comparative Analysis of Related Literature

Our findings align with several prior investigations that explored the relationship between skeletal malocclusion and airway volumes using CBCT. Nath et al. [23] conducted a retrospective study on 90 patients and found significantly larger pharyngeal airway volumes in Class III compared to Classes I and II, with Class I greater than Class II, consistent with our TPV and VPV results. Their use of similar landmarks (PNS to C4 for TPV) supports the comparability, though their smaller sample size (*n* = 90) may reduce statistical power compared to our cohort (*n* = 450). Pop et al. [10] assessed 90 orthodontic patients and found that TPV and oropharyngeal volumes were significantly larger in Class III patients compared to Class II patients, consistent with the current study's results, despite differences in TPV measurement landmarks (PNS to C2 vs. PNS to C4) and patient populations. Tseng et al. [11] reported that TPV, VPV, GPV, oropharyngeal, hypopharyngeal, and minimum cross-sectional airway volumes were significantly larger in Classes III and I patients compared to Class II patients, with nonsignificant differences between Classes III and I, corroborating the present findings. Shokri et al. [12] also found significantly larger nasopharyngeal volumes and minimum cross-sectional areas in Class III patients compared to Classes I and II, with Class I patients having greater volumes than Class II, aligning with this study's results. Alhammadi et al. [13], focusing on Classes I and II patients, reported greater nasopharyngeal, palatopharyngeal, and glossopharyngeal volumes in Class I compared to Class II, supporting the current study's findings for these classes. Hong et al. [14] found significantly larger upper, lower, and total pharyngeal airway volumes in Class III patients compared to Class I, consistent with the present study, though their exclusion of Class II patients limits direct comparison. Mardany et al. [15] analyzed pharyngeal airway volumes in 60 patients with Classes I, II, and III malocclusions using CBCT and found that Class III patients had significantly larger total pharyngeal and velopharyngeal volumes compared to Class I and Class II patients, with Class I patients exhibiting larger volumes than Class II. Mahmoud et al. [7] investigated the association between skeletal malocclusion, upper airway cross-sectional area, and airway volume in 80 patients using CBCT scans. Their results showed that Class III patients had significantly larger total pharyngeal and velopharyngeal airway volumes compared to Classes I and II patients, and Class I patients had larger volumes than Class II, consistent with the current study [7]. Elagib et al. [16] assessed pharyngeal airway volumes in 120 Korean adolescents and found that Class III patients had significantly larger total pharyngeal and velopharyngeal volumes compared to Class II patients, with Class I patients showing intermediate volumes, supporting the current study's findings. However, their focus on adolescents and inclusion of cervical vertebral maturation stages may introduce variability due to growth-related changes, unlike the adult population in the present study [16].

In contrast, some studies reported no significant relationship between airway volumes and skeletal malocclusion, differing from the current findings. Jadhav et al. [17] found significantly larger upper, middle, and lower nasopharyngeal airway volumes in Class III patients compared to Classes I and II, and in Class I compared to Class II, but no significant difference in total nasopharyngeal airway volume across skeletal classes. This discrepancy may stem from differences in measurement landmarks, as Jadhav et al. measured from the pituitary fossa to the upper epiglottis, whereas the current study used the PNS to C4, potentially capturing a broader airway segment that revealed significant differences. Di Carlo et al. [18] reported no significant relationship between nasopharyngeal, velopharyngeal, and oropharyngeal volumes and skeletal malocclusion, though they noted larger TPV and minimum cross-sectional areas in Class III patients compared to Classes I and II, partially aligning with the current study. Kula et al. [19] found no significant correlation between nasal cavity, nasopharyngeal, oropharyngeal, and hypopharyngeal volumes and skeletal malocclusion in 83 children aged 5–13 years, contrasting with the current study's findings. The primary reason for this discrepancy is likely the difference in patient age, as pediatric airway measurements may be influenced by growth and adenoid hypertrophy [20].

Additional studies provide further context. Brito et al. [24] conducted a 3D CBCT study focusing on Class II malocclusion patterns (*n* = 60) and found that subdivisions of Class II (e.g., Division 1 vs. Division 2) had varying airway volumes, with Division 1 showing smaller oropharyngeal volumes compared to controls, supporting our finding of reduced airway volumes in Class II patients. Their focus on Class II subgroups, rather than comparing all malocclusion classes, limits direct comparison but highlights the heterogeneity within Class II. Kose et al. [25] examined 75 children and found that Class II malocclusion was associated with smaller airway volumes, particularly when adenoid vegetation was present, aligning with our Class II findings. Their pediatric population and inclusion of paranasal sinus variations and adenoid hypertrophy introduce additional variables not assessed in our adult cohort, which may explain differences in the magnitude of airway volume reductions.

### 4.2. Factors Explaining Discrepancies

Discrepancies between our findings and studies like Jadhav et al. [17], Di Carlo et al. [18], and Kula et al. [19] may be attributed to several factors. First, sample size impacts statistical power: our study's large cohort (*n* = 450) enhances the ability to detect significant differences compared to smaller studies (e.g., *n* = 90 in Di Carlo et al. [18], *n* = 83 in Kula et al.[19]). Second, measurement landmarks vary: our use of PNS to C4 for TPV captures a broader airway segment compared to pituitary fossa to epiglottis [17] or PNS to C2 [10], potentially revealing larger differences. Third, patient age influences results: studies in children (e.g., Kula et al. [19], Kose et al. [25]) face variability from craniofacial growth and adenoid hypertrophy, whereas our adult cohort (17–39 years) reflects stable anatomy [19, 25]. Fourth, CBCT positioning affects measurements: our standing position maintains natural airway posture, while supine positioning in some studies (e.g., Di Carlo et al. [18]) may cause soft tissue collapse, reducing volumes [18]. Finally, classification criteria differ: our strict ANB ranges (Class I: 1°–4°; Class II: >4°; Class III: <0°) ensure precise group differentiation, unlike broader ranges in some studies (e.g., Di Carlo et al. [18]), which may dilute differences [18].

### 4.3. Clinical Implications

The significant relationship between skeletal malocclusion and airway volumes has important clinical implications, particularly for orthodontic and airway-related treatment planning. The finding that Class III patients have larger TPV and VPV compared to Classes I and II patients suggests that mandibular prognathism may be associated with a reduced risk of airway obstruction, potentially lowering the likelihood of conditions like OSA. Conversely, Class II patients, with significantly smaller TPV, VPV, and GPV, may be at higher risk for airway narrowing and OSA, necessitating careful evaluation.

This study has several limitations that must be acknowledged. The cross-sectional design of this study precludes establishing causality between skeletal malocclusion and airway volumes, as it captures data at a single point in time and cannot account for changes over time due to growth, aging, or orthodontic treatment. Longitudinal studies would be necessary to assess how airway volumes evolve and whether interventions alter these relationships. Additionally, the study did not include functional airway assessments, such as airflow dynamics or resistance, which are critical for understanding the clinical significance of volumetric differences, particularly in relation to OSA risk. Sleep-related metrics, such as apnea–hypopnea index (AHI) or polysomnography data, were not evaluated, limiting the ability to directly link airway volume findings to OSA severity or other sleep-disordered breathing conditions. Furthermore, patient-specific variables, such as body mass index (BMI), neck circumference, or soft tissue characteristics (e.g., tongue size or fat deposition), were not measured, despite their known influence on airway dimensions and OSA risk. These unmeasured variables could confound the observed relationships between skeletal malocclusion and airway volumes, as higher BMI or excessive soft tissue may disproportionately affect airway patency in certain malocclusion classes, particularly Class II. Future research should incorporate these functional and patient-specific factors to provide a more comprehensive understanding of airway-related implications in skeletal malocclusion

## 5. Limitations

This study's cross-sectional design precludes causality assessment, as it captures data at a single time point, limiting insights into changes from growth, aging, or treatment. Longitudinal studies are needed to evaluate airway volume evolution. The absence of functional airway assessments (e.g., airflow dynamics, AHI) limits direct linkage to OSA severity. Patient-specific factors like BMI, neck circumference, or soft tissue characteristics were not measured, despite their influence on airway dimensions [26–29]. Future research should incorporate these variables to better understand airway-related implications in skeletal malocclusion.

## 6. Conclusion

This study, despite its limitations, confirms a significant relationship between skeletal malocclusion and airway volumes. The results demonstrated that the mean TPV and VPV were significantly greater in Class III patients compared to Classes II and I patients. Additionally, Class I patients exhibited significantly larger TPV and VPV compared to Class II patients. The GPV was significantly larger in Class III patients than in Class II patients, with no significant difference between Class I and either Class II or III. These findings have practical implications for orthodontic diagnosis and treatment planning, particularly for Class II patients who may be at higher risk for airway narrowing and OSA due to smaller airway volumes. Clinicians should consider incorporating CBCT-based airway assessments into the diagnostic process for Class II patients to identify those at risk for OSA or airway-related issues. Orthodontic interventions, such as mandibular advancement or maxillary expansion, could be prioritized to optimize airway dimensions alongside dental alignment, potentially reducing OSA risk and improving respiratory health in these patients.

## Data Availability

The datasets supporting the findings of the current study are available from the corresponding author upon reasonable request.
